# Complete genome sequence and lifestyle of black-pigmented *Corynebacterium aurimucosum *ATCC 700975 (formerly *C. nigricans *CN-1) isolated from a vaginal swab of a woman with spontaneous abortion

**DOI:** 10.1186/1471-2164-11-91

**Published:** 2010-02-05

**Authors:** Eva Trost, Susanne Götker, Jessica Schneider, Susanne Schneiker-Bekel, Rafael Szczepanowski, Alexandra Tilker, Prisca Viehoever, Walter Arnold, Thomas Bekel, Jochen Blom, Karl-Heinz Gartemann, Burkhard Linke, Alexander Goesmann, Alfred Pühler, Sanjay K Shukla, Andreas Tauch

**Affiliations:** 1 Institut für Genomforschung und Systembiologie, Centrum für Biotechnologie, Universität Bielefeld, Universitätsstraße 27, D-33615 Bielefeld, Germany; 2 CLIB Graduate Cluster Industrial Biotechnology, Centrum für Biotechnologie, Universität Bielefeld, Universitätsstraße 27, D-33615 Bielefeld, Germany; 3 Bioinformatics Resource Facility, Centrum für Biotechnologie, Universität Bielefeld, Universitätsstraße 25, D-33615 Bielefeld, Germany; 4 International NRW Graduate School in Bioinformatics and Genome Research, Centrum für Biotechnologie, Universität Bielefeld, Universitätsstraße 25, D-33615 Bielefeld, Germany; 5 Institut für Innovationstransfer an der Universität Bielefeld GmbH, Universitätsstraße 25, D-33615 Bielefeld, Germany; 6 Lehrstuhl für Genomforschung, Fakultät für Biologie, Universität Bielefeld, Universitätsstraße 27, D-33615 Bielefeld, Germany; 7 Lehrstuhl für Gentechnologie und Mikrobiologie, Fakultät für Biologie, Universität Bielefeld, Universitätsstraße 25, D-33615 Bielefeld, Germany; 8 Clinical Research Center, Marshfield Clinic Research Foundation, Marshfield, Wisconsin, USA

## Abstract

**Background:**

*Corynebacterium aurimucosum *is a slightly yellowish, non-lipophilic, facultative anaerobic member of the genus *Corynebacterium *and predominantly isolated from human clinical specimens. Unusual black-pigmented variants of *C. aurimucosum *(originally named as *C. nigricans*) continue to be recovered from the female urogenital tract and they are associated with complications during pregnancy. *C. aurimucosum *ATCC 700975 (*C. nigricans *CN-1) was originally isolated from a vaginal swab of a 34-year-old woman who experienced a spontaneous abortion during month six of pregnancy. For a better understanding of the physiology and lifestyle of this potential urogenital pathogen, the complete genome sequence of *C. aurimucosum *ATCC 700975 was determined.

**Results:**

Sequencing and assembly of the *C. aurimucosum *ATCC 700975 genome yielded a circular chromosome of 2,790,189 bp in size and the 29,037-bp plasmid pET44827. Specific gene sets associated with the central metabolism of *C. aurimucosum *apparently provide enhanced metabolic flexibility and adaptability in aerobic, anaerobic and low-pH environments, including gene clusters for the uptake and degradation of aromatic amines, L-histidine and L-tartrate as well as a gene region for the formation of selenocysteine and its incorporation into formate dehydrogenase. Plasmid pET44827 codes for a non-ribosomal peptide synthetase that plays the pivotal role in the synthesis of the characteristic black pigment of *C. aurimucosum *ATCC 700975.

**Conclusions:**

The data obtained by the genome project suggest that *C. aurimucosum *could be both a resident of the human gut and possibly a pathogen in the female genital tract causing complications during pregnancy. Since hitherto all black-pigmented *C. aurimucosum *strains have been recovered from female genital source, biosynthesis of the pigment is apparently required for colonization by protecting the bacterial cells against the high hydrogen peroxide concentration in the vaginal environment. The location of the corresponding genes on plasmid pET44827 explains why black-pigmented (formerly *C. nigricans*) and non-pigmented *C. aurimucosum *strains were isolated from clinical specimens.

## Background

In 2002, *Corynebacterium aurimucosum *was taxonomically described as new species within the genus *Corynebacterium*, with *C. minutissimum *as the nearest phylogenetic neighbour [1,2]. The phylogenetic and molecular taxonomic description was based on two isolates from human clinical specimens, one from an unknown source and one from blood cultures of a patient with bronchitis. *C. aurimucosum *is a non-lipophilic species, and growth is facultatively anaerobic. On Columbia blood agar colonies are generally slightly yellow in colour [1]. The isolation of *C. aurimucosum *from other human clinical sources has been reported, although very rarely. *C. aurimucosum *was detected in samples from patients with acute or chronic joint or bone infections [3,4], in infected diabetic foot wounds [5] and in a biopsy sample from a patient with rheumatoid arthritis [6]. Furthermore, *C. aurimucosum *16S rDNA was detected in a bacterial population collected in the entrance area of a clean room environment in the Johnson Space Center [7,8] and in dust samples taken from office rooms in buildings located in central Finland [9].

Moreover, unusual charcoal-black-pigmented variants of *C. aurimucosum *were isolated in the U.S.A. and Canada from female urogenital sources, mostly from vaginal and cervical swabs [10-12]. In 2001, Shukla and co-workers reported the characterization of strain CN-1 (ATCC 700975) that was isolated from a vaginal swab of a 34-year-old female who experienced a spontaneous abortion during month six of pregnancy [10]. Subsequently, additional isolates with similar phenotypic and genotypic characteristics were recovered from the genital tract of women who had complications during pregnancy [12,13], suggesting that this newly recognized corynebacterium might be an opportunistic pathogen in pregnant women [13]. According to their black pigmentation, the name *Corynebacterium nigricans *was proposed for these isolates, and CN-1 was selected as type strain of this species [14]. However, morphological and biochemical analyses and sequencing of 16S rRNA genes in a polyphasic study of black-pigmented coryneform bacteria from the Centers for Disease Control and Prevention (CDC) group FCG4 suggested that *C. nigricans *is a later synonym for *C. aurimucosum *[12].

In this study, we present the complete genome sequence and bioinformatics analysis of *C. aurimucosum *ATCC 700975 (*C. nigricans *CN-1) to obtain insights into the physiology and lifestyle of this potential urogenital pathogen. Strain CN-1 was originally isolated from a female who presented with the sudden onset of premature labor during month six of gestation. The medical history of the patient was unremarkable, but the fetus died during vaginal delivery. A vaginal sample was taken at the time of delivery and revealed moderate growth of a black-pigmented corynebacterium in conjunction with other normal vaginal flora [10].

## Results

### Pyrosequencing and annotation of the *C. aurimucosum *ATCC 700975 genome

**Figure 1:**
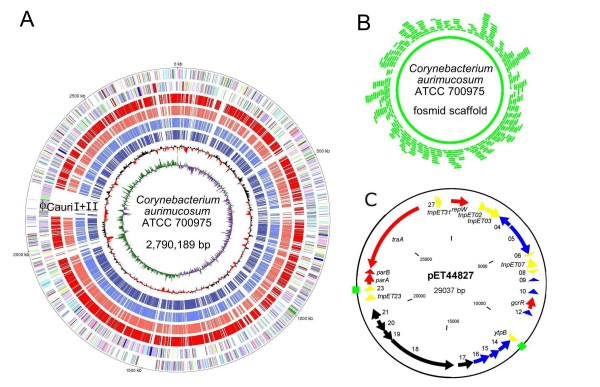
**The complete genome of the clinical isolate *C. aurimucosum *ATCC 700975**. **(A)**, Plot of the *C. aurimucosum *ATCC 700975 chromosome. The circles represent from the outside: circle 1, DNA base position; circles 2 and 3, predicted protein-coding sequences transcribed clockwise and anticlockwise, respectively; circles 4 to 7, genes encoding orthologous proteins in *C. diphtheriae *NCTC 13129, *C. jeikeium *K411, *C. urealyticum *DSM7109, and *C. kroppenstedtii *DSM44385; circle 8, G+C content plotted using a 10-kb window; circle 9, G/C skew plotted using a 10-kb window. The predicted protein-coding sequences are coloured according to their functional classification into the Clusters of Orthologous Groups of proteins [93]. The genomic position of the putative prophages ϕCauri I and ϕCauri II is marked. **(B)**, Fosmid scaffold of the *C. aurimucosum *ATCC 700975 chromosome. Individual fosmid clones used to build the genomic scaffold by terminal insert sequencing are represented as green segments. **(C)**, Genetic map of plasmid pET44827 detected in *C. aurimucosum *ATCC 700975. The predicted protein-coding regions are shown by arrows indicating the direction of transcription. A direct repeat region is indicated as green box. Colour code: black, non-ribosomal peptide synthetase (NRPS) gene region; yellow, transposase genes and repetitive sequences; red, genes involved in plasmid replication and maintenance; blue, genes encoding hypothetical proteins.

The complete genome sequence of the clinical isolate *C. aurimucosum *ATCC 700975 was determined by combining a whole-genome shotgun approach performed by pyrosequencing with a whole-genome scaffold generated by fosmid technology (Fig. 1A). A single run with the Genome Sequencer FLX System yielded 594,627 reads and a total number of 136,924,144 bases that were assembled into 73 large (≥ 500 bases) contigs. These assembled DNA sequences (2,736,233 bases) were combined with 215 Sanger reads performed on selected fosmid clones that covered the gaps between 70 chromosomal contigs. The resulting assembly of the DNA sequence data into a circular chromosome of 2,790,189 bp was consistent with the whole-genome scaffold deduced from 432 terminal insert sequences of randomly selected fosmid clones (Fig. 1B), corroborating the accuracy of the assembly process that was guided by the Consed program [15]. The gaps between the three remaining contigs (28,223 bases) were bridged by PCR, resulting in a circular contig of 29,037 bp in length. This assembled DNA sequence represents an endogenous plasmid of *C. aurimucosum *ATCC 700975, named pET44827 (Fig. 1C). The average gap size between the 70 chromosomal contigs was 1174 bp and 271 bp between the three plasmid contigs. Considering the final size of the *C. aurimucosum *ATCC 700975 genome, a 49-fold coverage was initially obtained by pyrosequencing. Relevant data of the *C. aurimucosum *ATCC 700975 genome project are summarized in Table 1.

**Table 1:** Features of the *C. aurimucosum *ATCC 700975 genome

Feature	Chromosome	Plasmid pET44827
Total size (bp)	2,790,189	29,037
G+C content (%)	60.6	53.3
No. of protein-coding sequences	2,531	20
No. of pseudogenes	4	8
Coding density (%)	88	64
Average gene length (bp)	973	928
No. of rRNAs	4 × 16S-23S-5S	0
No. of tRNAs	55	0
No. of transposase genes	85	5 (3 pseudogenes)
No. of CRISPR ^1^	98	0

^1^Abbreviation: Clustered Regularly Interspaced Short Palindromic Repeats

The circular chromosome of *C. aurimucosum *ATCC 700975 has a size of 2,790,189 bp and a mean G+C content of 60.6% (Fig. 1A), which is slightly below the value of 63.7% that was previously determined by high-performance liquid chromatography [1]. Gene prediction and annotation of the genome sequence were performed with the GenDB system [16] and resulted in the detection of 2,531 protein-coding regions on the *C. aurimucosum *chromosome. On plasmid pET44827, twenty protein-coding sequences and eight pseudogenes were detected (Fig. 1C). Furthermore, 55 tRNA genes were found by the tRNAscan-SE program [17], and four *rrn *operons were identified on the leading strands of the *C. aurimucosum *chromosome, one on the right and three on the left replichore.

### General architecture of the *C. aurimucosum *ATCC 700975 genome: moderate chromosomal reorganization in the main lineage of the genus *Corynebacterium*

**Figure 2:**
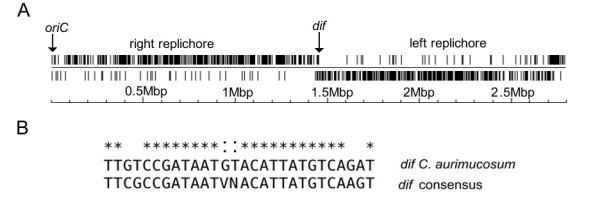
**Distribution of architecture imparting sequences in the chromosome of *C. aurimucosum *ATCC 700975 and nucleotide sequence of the deduced *dif *site**. **(A)**, Distribution of the architecture imparting sequences G(A/T/C)GGGGGA and (T/C)GGGGGAG on the leading and lagging strands of the *C. aurimucosum *ATCC 700975 chromosome. The deduced position of the *dif *locus at around 1.425 Mbp is indicated in the linear representation of the chromosome. The position of the origin of replication (*oriC*) is marked. **(B)**, Nucleotide sequence of the *dif *region in the *C. aurimucosum *ATCC 700975 chromosome. The 28-bp sequence is aligned with the consensus sequence of actinobacterial *dif *sites [20]. Identical nucleotides of the alignment are marked with asterisks. Matches to non-conserved nucleotides in the *dif *consensus sequence are specifically marked (:). Abbreviations: V = {A, C, G}; N = {A, C, G, T}.

The calculation of the G/C skew [(G-C)/(G+C)] [18] indicated a bi-directional replication mechanism of the *C. aurimucosum *ATCC 700975 chromosome (Fig. 1A). The origin of replication (*oriC*) was detected with Ori-Finder, a web-based tool for *oriC *prediction in bacterial genomes [19]. According to the presence and distribution of seven conserved DnaA boxes [TTATC(C/A)A(C/A)A], the *oriC *is located downstream of the *dnaA *(cauri_0001) coding region and has a computed length of 423 bp. The calculated G/C skew additionally indicated the presence of a putative *dif *region involved in replication termination [20] at the expected position of 180° from *oriC*, dividing the chromosome of *C. aurimucosum *ATCC 700975 in two replichores of similar size (Fig. 1A). For a more precise detection of a potential *dif *region, the distribution of the architecture imparting sequences G(A/T/C)GGGGGA and (T/C)GGGGGAG [21] was plotted on the leading and lagging strands of the *C. aurimucosum *ATCC 700975 chromosome (Fig. 2A). These octamers are present in 856 copies on the leading strands and in only 61 copies on the lagging strands of the *C. aurimucosum *ATCC 700975 chromosome. This characteristic distribution pattern of architecture imparting sequences revealed a putative *dif *region at around 1,425 kb of the chromosomal map (Fig. 2A). In accordance with this result, the respective DNA region of the *C. aurimucosum *ATCC 700975 chromosome contains a 28-bp sequence that showed striking similarity to the consensus sequence of actinobacterial *dif *sites [20] (Fig. 2B).

**Figure 3:**
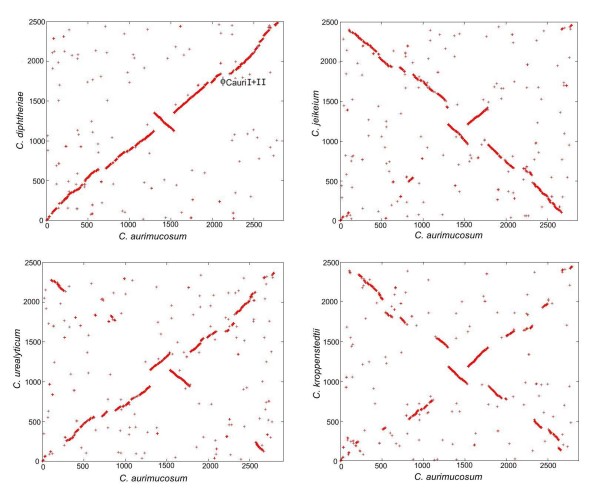
**Synteny between the chromosome of *C. aurimucosum *ATCC 700975 and those from *C. diphtheriae *NCTC 13129, *C. jeikeium *K411, *C. urealyticum *DSM7109, and *C. kroppenstedtii *DSM44385**. The graphs represent X-Y plots of dots forming syntenic regions between the chromosomes of pathogenic corynebacteria. Each dot represents a predicted *C. aurimucosum *ATCC 700975 protein having an ortholog in another corynebacterial genome with co-ordinates corresponding to the position of the respective coding region in each genome. Orthologous proteins were detected by reciprocal best BLASTP matches. The genomic position of the putative prophages ϕCauriI and ϕCauriII is marked in the synteny plot with *C. diphtheriae *NCTC 13129.

Synteny analysis by reciprocal best matches with BLASTP [22] revealed a conserved order of orthologous genes between the *C. aurimucosum *ATCC 700975 chromosome and other pathogenic corynebacteria, in particular with *C. diphtheriae *NCTC 13129 displaying two breakpoints of synteny at 1,298 kb and 1,537 kb of the *C. aurimucosum *chromosomal map (Fig. 3). The comparison between the gene order of *C. aurimucosum *ATCC 700975 and *C. jeikeium *K411, *C. urealyticum *DSM7109 and *C. kroppenstedtii *DSM44385 revealed additional breakpoints in the chromosomal organization (Fig. 3), which is consistent with the taxonomic position of the latter three species that are part of independent branches in the phylogenetic tree of the genus *Corynebacterium *[2]. The two breakpoints of synteny between the chromosomes of *C. aurimucosum *ATCC 700975 and *C. diphtheriae *NCTC 13129 are indicative of a symmetric inversion of a 239-kb chromosomal DNA segment (carrying the genes cauri_1172 to cauri_1381) around the terminus of replication. The detected inversion in the *C. aurimucosum *ATCC 700975 chromosome provides the first example of a moderate genetic reorganization in genomes of members from the main lineage of the genus *Corynebacterium*, since all hitherto completely sequenced genomes (*C. diphtheriae *NCTC 13129, *C. glutamicum *ATCC 13032, *C. efficiens *YS-314) displayed an overall synteny of their gene order [23].

Due to the symmetric inversion around the terminus of the *C. aurimucosum *ATCC 700975 chromosome, the preferential location of the respective genes on either the leading or lagging strands and their distance from the terminus region remained conserved after the chromosomal reorganization [24]. In general, 61% of the predicted *C. aurimucosum *ATCC 700975 genes are located on the leading strands of both replichores, while 39% are encoded on the lagging strands. It has been suggested that essentiality is the driving force for such type of gene-strand bias in bacterial genomes [25]. To detect candidate essential genes in the *C. aurimucosum *ATCC 700975 genome, the predicted gene repertoire was compared with that of *C. glutamicum *strain R [26], as genes shared by multiple genomes are more likely to be essential [27]. In addition, a high-density transposon mutagenesis approach in *C. glutamicum *R had already revealed 658 candidate essential genes in this strain, using complex medium and standard laboratory conditions for the selection of transposon mutants [28]. This number of candidate essential genes obviously represents an overestimate, at least due to polar effects caused by some transposon insertions [27]. The comparative genomics approach detected 352 coding regions of *C. aurimucosum *ATCC 700975 with high similarity to candidate essential genes from *C. glutamicum *R. Among this set of candidate essential genes, 78.8% are located on the leading strands of the *C. aurimucosum *ATCC 700975 chromosome, while 21.2% are encoded on the lagging strands, which is consistent with the aforementioned interpretation of gene-strand bias in bacterial genomes [25].

The annotation of the *C. aurimucosum *ATCC 700975 chromosome revealed the presence of two putative prophages. This observation is consistent with the lack of homology of the respective chromosomal region to the genomes of other pathogenic corynebacteria (Fig. 1A) and with a lack of synteny when compared with the genome sequence of *C. diphtheriae *NCTC 13129 (Fig. 3). The first prophage-like region (ϕCauriI) was detected adjacent to tRNA^Leu ^and tRNA^Gln ^genes of *C. aurimucosum *ATCC 700975 that may represent the integration site of ϕCauriI. This genome region has a size of about 20.7 kb and encodes 35 genes (cauri_1932 to cauri_1966). The second prophage-like region (ϕCauriII) is located directly adjacent to ϕCauriI and comprises 62 genes (cauri_1967 to cauri_2028) with a size of about 51.5 kb. Both prophage regions contain at their 3' ends genes encoding λ repressor-like transcription regulators (cauri_1965 and cauri_2027) and phage-related integrases (cauri_1966 and cauri_2028). Almost all genes of the prophage regions, with the exception of the regulatory genes, are encoded on the leading strand of the left replichore of the *C. aurimucosum *ATCC 700975 chromosome (Fig. 1A). The size difference between both prophage regions suggests that at least ϕCauriI is incomplete and a defective remnant of a formerly active corynephage.

Beside that, we detected with the CRISPRFinder program [29] an array of 98 clustered regularly interspaced short palindromic repeats (CRISPRs) and seven CRISPR-associated (*cas*) genes (cauri_0899 to cauri_0905) in the *C. aurimucosum *ATCC 700975 chromosome. The DNA repeats are 28 bp in length, separated by 33-bp spacers with variable nucleotide sequences and similar to repeat units of CRISPRs from *C. jeikeium *K411 [30], *C. urealyticum *DSM7109 [31] and *Nocardia farcinica *IFM 10152 [32]. CRISPR arrays and associated *cas *genes may provide acquired immunity against bacteriophages and other foreign genetic elements, with a specificity that is determined by sequence similarities between the spacers and foreign DNA sequences [33,34].

### Specific features of the carbohydrate metabolism of *C. aurimucosum *ATCC 700975: catabolism of aromatic amines and utilization of L-tartrate

The metabolic analysis of the *C. aurimucosum *ATCC 700975 genome was performed with the computer program CARMEN that provided an automatic mapping of genome annotations on manually curated metabolic pathway maps [35]. These data were combined with results from a comparative genomics approach performed with the bioinformatics tool EDGAR, resulting in the detection of orthologous genes in different corynebacterial genomes and their classification as core genes or species-specific genes, so-called singletons [36]. A comparison of the gene content of pathogenic corynebacteria revealed a putative core genome consisting of 1,048 genes that are common in *C. aurimucosum*, *C. diphtheriae*, *C. jeikeium*, *C. urealyticum*, and *C. kroppenstedtii*. This value is very similar to data deduced previously from the comparison of gene contents in pathogenic and non-pathogenic corynebacteria [26,30]. The comparative genome analysis with EDGAR revealed also that *C. aurimucosum *ATCC 700975 contains 443 genes that are specific for this chromosome. The functional evaluation of the detected singletons provided characteristic features of the metabolism and lifestyle of *C. aurimucosum *ATCC 700975 that are described in more detail below. As the specific gene repertoire of a bacterium should in principle be shaped by the environmental conditions in the natural habitat and as we observed similarities to the gene repertoire of gut microbes, the annotation data point towards the human gut as a natural source for *C. aurimucosum *strains, which is consistent with the recent single report of a detection of this organism in a human fecal sample [37].

**Figure 4:**
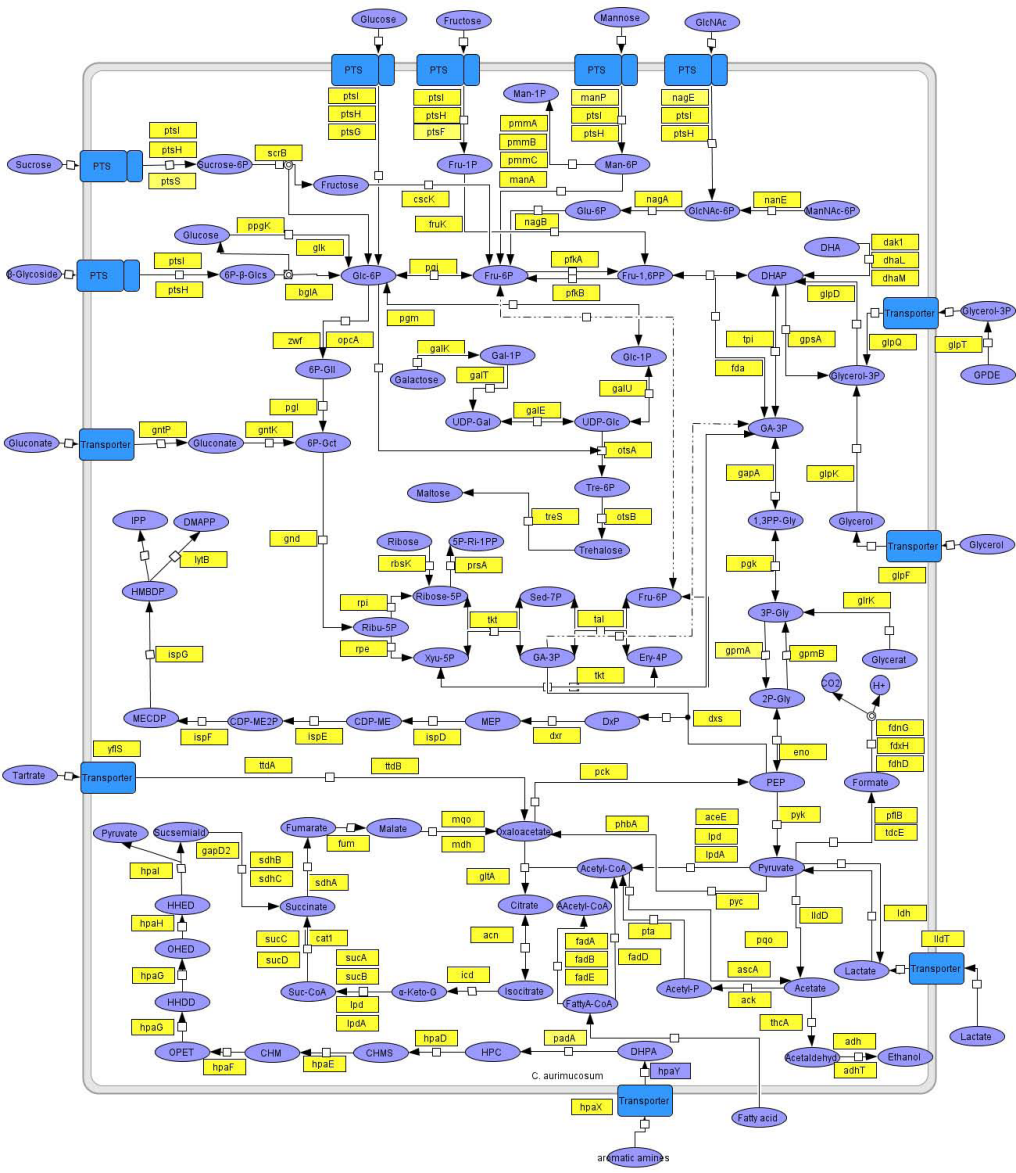
**Reconstruction of pathways involved in the central metabolism of *C. aurimucosum *ATCC 700975**. The metabolic reconstruction of the carbohydrate metabolism was facilitated by using manually curated pathway maps in conjunction with the bioinformatics tool CARMEN. The resulting reconstruction was visualized with the CellDesigner software (version 3.2). Genes encoding transporters or enzymes involved in carbohydrate uptake and metabolism are shown in yellow boxes. Key metabolites are indicated by purple circles. Abbreviations for metabolites are as follows: α-Keto-G, α-ketoglutarate; β-Glcs, β-glycoside; 1,3PP-Gly, glycerate-1,3-bisphosphate; 2P-Gly, 2-phosphoglycerate; 3P-Gly, 3-phosphoglycerate; 5P-Ri-1PP, 5-phospho-ribose-1-diphosphate; 6P-β-Glcs, 6-phospho-β-glycoside; 6P-Gct, 6-phosphogluconate; 6P-Gll, 6-phosphogluconolactone; Aacetyl-CoA, acetoacetyl-CoA; Acetyl-P, acetyl-phosphate; CDP-ME, 4-(cytidine 5'-diphospho)-2-*C*-methyl-D-erythritol; CDP-ME2P, 2-phospho-4-(cytidine 5'-diphospho)-2-*C*-methyl-D-erythritol; CHM, 5-carboxymethyl-2-hydroxy-muconic acid; CHMS, 5-carboxymethyl-2-hydroxy-muconic semialdehyde; DHA, dihydroxyacetone; DHAP, dihydroxyacetone phosphate; DMAPP, dimethylallyl diphosphate; DXP, 1-deoxy-D-xylulose 5-phosphate; Ery-4P, erythrose-4-phosphate; FattyA-CoA, fatty acyl-CoA; Fru-1,6PP, fructose-1,6-bisphosphate; Fru-1P, fructose-1-phosphate; Fru-6P, fructose-6-phosphate; GA-3P, glyceraldehyde-3-phosphate; Gal-1P, galactose-1-phosphate; Glc-1P, glucose-1-phosphate; Glc-6P, glucose-6-phosphate; GlcNAc, *N*-acetylglucosamine; GlcNAc-6P, *N*-acetylglucosamine-6-phosphate; Glu-6P, glucosamine-6-phosphate; Glycerol-3P, glycerol-3-phosphate; GPDE, glycerophosphodiester; HHDD, 2-hydroxy-hept-2,4-diene-1,7-dioic acid; HHED, 2,4-dihydroxy-hept-2-ene-1,7-dioic acid; HMBDP, 1-hydroxy-2-methyl-2-butenyl 4-diphosphate; HPC, homoprotocatechuate; IPP, isopentenyl diphosphate; Man-1P, mannose-1-phosphate; Man-6P, mannose-6-phosphate; ManNAc-6P, *N*-acetyl-mannosamine-6-phosphate; MECDP, 2-*C*-methyl-D-erythritol 2,4-cyclodiphosphate; MEP, 2-*C*-methyl-D-erythritol 4-phosphate; OHED, 2-oxo-hept-3-ene-1,7-dioic acid; OPET, 5-oxo-pent-3-ene-1,2,5-tricarboxylic acid; PEP, phosphoenolpyruvate; Ribose-5P, ribose-5-phosphate; Sed-7P, sedoheptulose-7-phosphate; Ribu-5P, ribulose-5-phosphate; Suc-CoA, succinyl-CoA; Sucrose-6P, sucrose-6-phosphate; Sucsemiald, succinate semialdehyde; Tre-6P, trehalose-6-phosphate; UDP-Gal, UDP-galactose; UDP-Glc, UDP-glucose; Xyu-5P, xylulose-5-phosphate.

Analysis of the central carbohydrate metabolism of *C. aurimucosum *ATCC 700975 revealed the complete gene set for glycolysis and the pentose phosphate pathway (Fig. 4). The chromosome of *C. aurimucosum *ATCC 700975 provides a phosphoenolpyruvate carboxykinase (*pck*) and a fructose-1,6-bisphosphatase (*pfkB*) function for gluconeogenesis. The anaplerotic reaction of the tricarboxylic acid (TCA) cycle is mediated by pyruvate carboxylase (*pyc*). In contrast to other sequenced pathogenic corynebacteria that all lack the *sucCD *genes coding for succinyl-CoA synthetase, the TCA cycle of *C. aurimucosum *ATCC 700975 is complete (Fig. 4). It has been suggested that a putative succinyl-CoA:CoA transferase encoded by the *cat1 *gene can replace the missing succinyl-CoA synthetase activity in pathogenic corynebacteria [30,38]. Besides *sucCD *(cauri_2063 and cauri_2062), the *C. aurimucosum *ATCC 700975 chromosome also carries the respective *cat1 *gene (cauri_2148). On the other hand, the *C. aurimucosum *ATCC 700975 genome lacks the *aceAB *genes for the glyoxylate bypass within the TCA cycle. The lack of *aceAB *was previously also observed in the genome sequence of *C. diphtheriae *NCTC 13129 [38].

**Figure 5:**
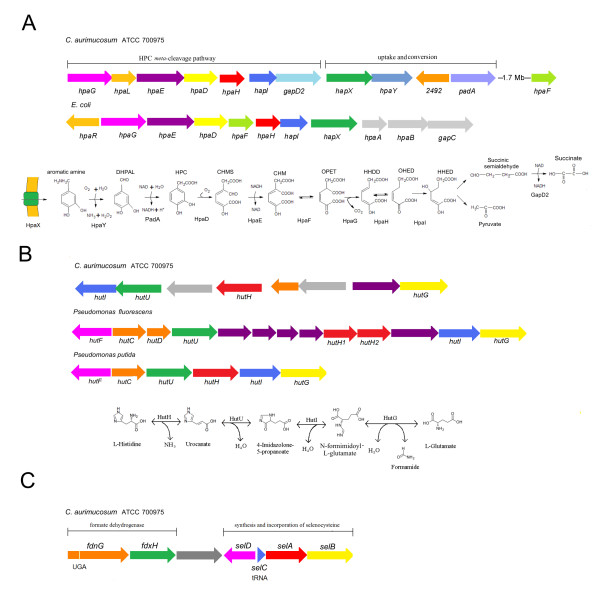
**Specific features of the *C. aurimucosum *ATCC 700975 metabolism detected by the analysis of singletons**. **(A)**, Genes and pathway for the catabolism of aromatic amines. Genetic maps of the *hpa *gene clusters encoding the HPC *meta*-cleavage pathway in *C. aurimucosum *and *E. coli *are presented. Genes with similar colour participate in the same enzymatic step of the pathway. The arrows show the direction of gene transcription. Abbreviations in the biochemistry of the pathway: DHPAL, 3,4-dihydroxyphenylacetaldehyde; HPC, homoprotocatechuate; CHMS, 5-carboxymethyl-2-hydroxy-muconic semialdehyde; CHM, 5-carboxymethyl-2-hydroxy-muconic acid; OPET, 5-oxo-pent-3-ene-1,2,5-tricarboxylic acid; HHDD, 2-hydroxy-hept-2,4-diene-1,7-dioic acid; OHED, 2-oxo-hept-3-ene-1,7-dioic acid; HHED, 2,4-dihydroxy-hept-2-ene-1,7-dioic acid. The enzymes are: HpaX, transport protein; HpaY, monoamine oxidase; PadA, phenylacetaldehyde dehydrogenase; HpaD, HPC 2,3-dioxygenase; HpaE, CHMS dehydrogenase; HpaF, CHM isomerase; HpaG, OPET decarboxylase; HpaH, hydratase; HpaI, HHED aldolase; GabD2, succinic semialdehyde dehydrogenase. **(B)**, Genes and pathway for the catabolism of L-histidine. Genetics maps of the *hut *gene clusters coding for histidine catabolism in *C. aurimucosum *and *Pseudomonas *species. Genes with similar colour participate in the same enzymatic step of the pathway. The enzymes of the *hut *pathway are: HutH, histidine ammonia-lyase; HutU, urocanate hydratase; HutI, imidazolonepropionase; HutG, formimidoylglutamase. **(C)**, Chromosomal gene region for selenol formate dehydrogenase and selenocysteine synthesis and incorporation in *C. aurimucosum *ATCC 700975. The UGA (opal) stop used for recoding the *fdnG *gene is indicated. The *selC *gene encodes the specific tRNA^Sec^.

The functional analysis of singletons in the *C. aurimucosum *ATCC 700975 genome sequence resulted in the detection of a distinct gene cluster for the uptake and degradation of aromatic amines (cauri_2491 to cauri_2501) (Fig. 4; Fig. 5A). *C. aurimucosum *ATCC 700975 can apparently take up aromatic amines by a specific permease encoded by *hpaX*. The subsequent transformation into ammonia and the corresponding aromatic acid is catalyzed by amine oxidase (*hpaY*) and phenylacetaldehyde dehydrogenase (*padA*). The resulting metabolite is converted into pyruvate and succinic semialdehyde *via *the homoprotocatechuate (HPC) *meta*-cleavage degradation route (Fig. 5A). The *hpa *gene cluster encoding the respective enzymes of this pathway consists of seven genes (*hpaGLEDHI *and *gabD2*). The *gabD2 *gene is required for the enzymatic dehydrogenation of succinic semialdehyde to succinate that can enter the TCA cycle (Fig. 4). The *hpaF *function necessary to convert 5-carboxymethyl-2-hydroxy-muconic acid (CHM) into 5-oxo-pent-3-ene-1,2,5-tricarboxylic acid (OPET) is encoded elsewhere in the *C. aurimucosum *ATCC 700975 chromosome (cauri_0890). Two regulatory genes (*hpaL *and cauri_2492) are apparently involved in controlling the expression of the HPC *meta*-cleavage pathway and the uptake and conversion of aromatic amines. The *hpa *gene cluster of *C. aurimucosum *ATCC 700975 is similar to a previously reported cluster for the uptake and degradation of aromatic acids in *E. coli *[39] (Fig. 5A). Aromatic compounds may constitute a carbon source for gut microbes, as they are not only abundant in soil and water, but are also present in the gastrointestinal tract, where they mostly derive from plant constituents. As the HPC 2,3-dioxygenase (HpaD) requires molecular oxygen, utilization of the HPC *meta*-cleavage pathway is restricted to distinct intestinal habitats, for instance ones in close proximity to epithelial cells, where oxygen molecules might pass from the blood to the microbes attached to the epithelium [40]. Accordingly, *C. aurimucosum *ATCC 700975 is probably able to utilize aromatic amines as carbon and energy sources when living in an aerobic environment, such as the peripheral areas of the human gut system.

Furthermore, the *C. aurimucosum *ATCC 700975 chromosome contains three singletons (cauri_2300 to cauri_2302) that are involved in the uptake and degradation of L-tartrate (Fig. 4). Uptake of L-tartrate is mediated by a tartrate/succinate antiporter encoded by *yflS*. The subsequent degradation to oxaloacetate is catalyzed by an (oxygen-labile) L-tartrate dehydratase (*ttdAB*). The respective gene cluster is homologous to previously described genes from *E. coli *[41,42]. Tartaric acid is found in many fruits and vegetables and is also a product of microbial metabolism in the gut ecosystem that enhances the ability of the normal gut microflora to reduce the intestinal colonization by *Salmonella *species and other enteropathogens [43]. L-tartrate is the most widely distributed isomer and can be degraded under aerobic and anaerobic conditions by a variety of bacteria [41]. However, *E. coli *can utilize L-tartrate only as reducible substrate for supporting anaerobic growth on the oxidizable co-substrate glycerol [41]. The *C. aurimucosum *ATCC 700975 chromosome also encodes a transporter for the uptake of glycerol (*glpF*) as well as glycerol-3-phosphate (*glpT*), gluconate (*gntP*) and lactate (*lldT*) transporters (Fig. 4). Furthermore, five genes coding for components of the bacterial phosphotransferase system (PTS) and two additional enzyme II genes (*manP*, *nagE*) encoding proteins of the fructose, glucose and glucoside (sub)families of PTS permeases [44] were identified in the chromosome of *C. aurimucosum *ATCC 700975 (Fig. 4). This gene repertoire, associated with the central metabolism of *C. aurimucosum *ATCC 700975, suggested that this bacterium is very flexible in the utilization of different carbon and energy sources, under both aerobic and anaerobic growth conditions.

### Specific features of the amino acid metabolism of *C. aurimucosum *ATCC 700975: L-histidine catabolism and utilization of selenocysteine in formate dehydrogenase

According to the genome annotation, all known pathways for the biosynthesis of standard proteinogenic amino acids are present in the *C. aurimucosum *ATCC 700975 chromosome. The detection of singletons with EDGAR revealed that *C. aurimucosum *ATCC 700975 encodes a complete histidine utilization (*hut*) pathway for the conversion of histidine to glutamate (Fig. 5B). Four gene products are directly involved in histidine catabolism: The first reaction is catalyzed by histidine ammonia-lyase (*hutH*; cauri_1023), followed by the conversion of the resulting urocanate to 4-imidazolone-5-propanoate *via *urocanate hydratase (*hutU*; cauri_1025). The next catabolic step generates *N*-formimidoyl-L-glutamate by imidazolonepropionase (*hutI*; cauri_1026) that is finally hydrolyzed into L-glutamate and formamide by formimidoylglutamase (*hutG*; cauri_1019). Similar gene regions are known from saprophytic bacteria, such as *Pseudomonas *species, that take up and utilize L-histidine when growing in the plant environment [45,46] (Fig. 5B).

Regarding the proposal of *C. aurimucosum *ATCC 700975 as urogenital pathogen in pregnant women, it is interesting to note that the female histidine metabolism is affected during pregnancy, resulting in the excretion of large amounts of histidine in the urine [47,48]. Histidine excretion is largely dependent on the composition of the diet and is generally higher during the second half of gravidity [47]. Although little information regarding the utilization by bacteria of L-histidine from human sources is available, the human gut microbe *Fusobacterium varium *was shown to use L-histidine as carbon source for growth [49]. According to the presence of a complete *hut *pathway, *C. aurimucosum *ATCC 700975 is apparently able to utilize L-histidine as carbon and/or nitrogen source, which might be beneficial for bacterial growth when colonizing the urogenital tract of pregnant women.

Moreover, the *C. aurimucosum *ATCC 700975 chromosome contains the *selABD *genes and the tRNA^Sec ^gene *selC *for the synthesis of the twenty-first proteinogenic amino acid selenocysteine (Sec) and its incorporation into selenoproteins (Fig. 5C). Selenocysteine is specifically incorporated into proteins by a cotranslational process that is directed by the UGA stop codon and termed recoding [50]. The first step during selenocysteine synthesis requires esterification of L-serine to the unique tRNA^Sec ^by L-seryl-tRNA synthetase (*serS*, cauri_2392). The seryl-tRNA^Sec ^is then converted to selenocysteyl-tRNA^Sec ^by selenocysteine synthetase (*selA*, cauri_0967), using selenophosphate as selenium donor, which in turn is synthesized by selenophosphate synthetase (*selD*, cauri_0966). The anticodon of the specific tRNA^Sec ^matches with an internal UGA stop codon in mRNAs from selenoprotein genes [51]. Furthermore, a specific elongation factor encoded by *selB *(cauri_0968) is required to recognize SECIS (selenocysteine insertion sequence) elements in the mRNA, such that the ribosome identifies the internal UGA stop codon as codon for selenocysteine. Translation of selenoprotein mRNAs then ends at the next in-frame stop codon to give a full-size selenoprotein [50,51].

Interestingly, the *fdnG *and *fdxH *genes (cauri_0962 and cauri_0964) encoding subunits of a formate dehydrogenase are arranged in a cluster with coding regions for the synthesis and incorporation of selenocysteine (Fig. 5C). This gene order is highly conserved in bacterial genomes that code for the incorporation of selenocysteine into proteins. Formate dehydrogenase is a heterogeneous group of enzymes that catalyze the decomposition of formate to carbon dioxide and contribute to mixed acid fermentation. Some formate dehydrogenase enzymes can contain an intrinsic selenocysteine residue [52]. The *fdnG *coding region of *C. aurimucosum *ATCC 700975 includes an internal UGA stop codon, suggesting that a formate dehydrogenase of the selenoenzyme type is encoded by this DNA region. The *C. aurimucosum *ATCC 700975 chromosome carries an additional gene for a standard (thiol) formate dehydrogenase (*fdhD*, cauri_0426) that is located adjacent to a putative formate transporter gene (cauri_0425). The utilization of the selenoenzyme form of formate dehydrogenase may enhance the adaptability of *C. aurimucosum *ATCC 700975 to changing environments, due to the strikingly different pH-dependence when compared with thiol forms, with selenols being active at much lower pH [53]. This biochemical feature of selenoenzymes is remarkable when considering the lifestyle of *C. aurimucosum *ATCC 700975, since acidophilic lactobacilli create a low-pH environment in the human vagina by producing lactic acid [54].

### Cell surface structures of *C. aurimucosum *ATCC 700975: synthesis of a SpaH-type pilus and potential in biofilm formation

**Figure 6:**
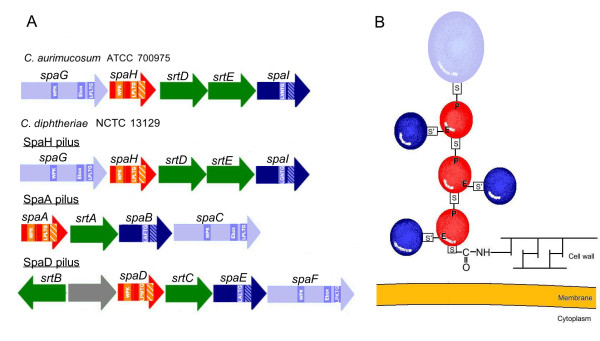
**The putative SpaH-like pilus of *C. aurimucosum *ATCC 700975**. **(A)**, Genes clusters involved in the synthesis of adhesive pili in *C. aurimucosum *ATCC 700975 and *C. diphtheriae *NCTC 13129. Colour code: green, sortases required for the assembly of the pilus; red, major pilin; dark blue, minor pilin; light blue, tip protein; grey, protein of unknown function. Specifically marked are: pilin boxes (WPK), E-boxes (Ebox), sorting signals (LPXTG), hydrophobic domains in major pilins (hatched boxes), and charged tails in minor pilins (hatched boxes). **(B)**, Model representation of the corynebacterial SpaH-like pilus. The pilus is covalently anchored to the corynebacterial cell wall [55]. Sorting motifs (S) and pilin motifs (P) used for the pilus assembly, as well as E-boxes (E) and sorting signals used for anchoring of the minor pilins (S') are marked.

The *C. aurimucosum *ATCC 700975 genome codes for 438 proteins containing predicted signal peptides for either the general secretory (sec) or the twin-arginine translocation (tat) pathway for protein secretion. Three secreted proteins of *C. aurimucosum *ATCC 700975 showed homology to subunits of the adhesive SpaH pilus from *C. diphtheriae *NCTC 13129 [55]. Adhesive pili are covalently anchored to the corynebacterial cell wall by a transpeptidylation mechanism, requiring a C-terminal sorting signal with a conserved LPXTG motif [56], and can mediate the adherence to host tissues and other bacterial cells [57]. The chromosome of *C. aurimucosum *ATCC 700975 contains two genes, *srtD *and *srtE *(cauri_0189 and cauri_0190), encoding putative sortases involved in the assembly of a SpaH-like pilus. The housekeeping sortase of *C. aurimucosum *ATCC 770975 is encoded elsewhere in the chromosome (*srtB*, cauri_2420). The *srtDE *genes of *C. aurimucosum *ATCC 700975 are part of a DNA region also comprising the *spaHIG *genes (cauri_0187, cauri_0188, cauri_0191) that encode surface-anchored proteins with C-terminal sorting motifs (Fig. 6A). These proteins showed similarity to fimbrial subunits from *C. diphtheriae *NCTC 13129 involved in the sortase-mediated formation of the SpaH pilus [55]. The SpaH protein represents the major pilin of this pilus type and, along with the minor pilin SpaI, forms the pilus shaft, whereas the SpaG protein is located at the tip of the pilus. According to the detected homology between the corynebacterial SpaHIG proteins, it is likely that *C. aurimucosum *ATCC 700975 expresses an adhesive pilus with structural similarity to the SpaH pilus of *C. diphtheriae *NCTC 13129 (Fig. 6B). This pilus structure can mediate the initial adhesion of *C. aurimucosum *ATCC 700975 to host tissues particularly during vaginal infection and/or colonization [58].

Further analysis of the genome sequence revealed that *C. aurimucosum *ATCC 700975 codes for a putative accumulation-associated protein (*aap*, cauri_1006). Bioinformatics analysis displayed five G5 domains within the amino acid sequence of Aap that can mediate the formation of biofilms by recognizing and binding to *N*-acetylglucosamines present in the peptidoglycan of the bacterial cell wall [59]. Moreover, the C-terminal segment of Aap contains a sorting motif (LANTG) for anchoring of the protein to the corynebacterial cell wall. The secretion mechanism of Aap remains unknown, as the protein is devoid of a typical signal peptide. The Aap protein of *C. aurimucosum *ATCC 700975 is also characterized by a C-terminal multiple repeat unit of six amino acids (PDEPGK), the number of which may vary among different isolates and during the course of an infection, as described for members of the biofilm-associated protein (Bap) family [60,61]. Variations in the Aap protein sequence may help the bacterium to evade the immune response of the host, as observed in the case of the alpha C protein of group B streptococci [62]. The Aap protein of *C. aurimucosum *ATCC 700975 shares structural similarity with the biofilm-associated protein Bap of *Staphylococcus aureus *[63] and the accumulation-associated protein Aap of *Staphylococcus epidermidis *[64]. Both staphylococcal proteins are located on the cell surface and implicated in the formation of biofilms. Cell aggregation and biofilm formation by Aap requires proteolytic processing of the protein by endogenous or host-derived proteases [64]. In the latter case, host proteins can directly induce biofilm formation upon infection, thereby enabling the pathogen to evade the clearance by phagocytes. Both the Aap protein and the SpaH-type pilus apparently represent prominent protein factors on the surface of *C. aurimucosum *ATCC 700975 cells that are most likely involved in adhesion and colonization processes and in the formation of biofilms.

### The gene repertoire of plasmid pET44827: synthesis of the characteristic black pigment of *C. aurimucosum *ATCC 700975

Assembly of the *C. aurimucosum *ATCC 700975 genome sequence revealed the presence of the endogenous plasmid pET44827 (Fig. 1C). The average G+C content of 53.3% is below the value of 60.6% determined for the *C. aurimucosum *ATCC 700975 chromosome, indicating the acquisition of pET44827 by horizontal gene transfer. Coding regions displaying amino acid sequence similarities with proteins of known functions and providing information about structural features of pET44827 and the physiological role of the deduced proteins are presented below.

Replication of pET44827 is presumably mediated by a replication initiator protein of the IncW type (*repW*) that showed 89% amino acid sequence identity to a homologous counterpart on the multiresistance plasmid pTP10 from *C. striatum *M82B [65]. This indicates that pET44827 replicates by the theta mechanism, in contrast to other corynebacterial plasmids belonging to the pNG2 family that replicate *via *the rolling-circle mode [66]. Additionally, pET44827 encodes a putative resolvase (*gcrR*) ensuring the stability of the plasmid during segregation. The putative resolvase of pET44827 contains a conserved N-terminal catalytic domain of the serine recombinase family and a C-terminal DNA binding domain [67]. However, the *gcrR *gene region lacks the typical DNA sequence repeats described for the homologous resolvase gene of pTP10 from *C. striatum *M82B [65]. Furthermore, pET44827 encodes a putative relaxase encoded by *traA *with 52% similarity to the relaxase of plasmid pREA400 from *Rhodococcus erythropolis *AN12 [68]. Two genes located upstream of *traA *were identified as *parA *and *parB*, showing similarity to class Ib plasmid partitioning genes present on the pNG2-family plasmid pTET3 from *C. glutamicum *LP-6 [69]. Therefore, pET44827 seems to be an unusual corynebacterial plasmid that contains characteristic features of the pNG2 family that are combined with a replication initiator protein of the IncW type.

The most prominent feature of pET44827 is a region composed of five genes, including *orf18 *coding for a putative non-ribosomal peptide synthetase (NRPS) (Fig. 1C). The G+C content of the NRPS gene region is about 47%, which is below the mean value detected for pET44827 (Table 1). As the NRPS gene region is flanked by a remnant of a transposase gene (*tnpET23*), it might have been integrated into the backbone of pET44827 by a transpositional recombination event. The deduced gene product of *orf18 *shares 52% similarity with the single module type NRPS BpsA from *Streptomyces lavendulae *[70] and 51% similarity with the NRPS IndC from the phytopathogen *Erwinia chrysanthemi *[71]. Both NRPSs catalyze the modular synthesis of the blue 3,3'-bipyridyl pigment indigoidine. The predicted NRPS of pET44827 comprises ten of the eleven highly conserved amino acid sequence motifs of NRPS proteins, only lacking a typical N-terminal condensation domain. Likewise, the IndC protein from *E. chrysanthemi *does not contain a condensation domain, suggesting that the final condensation step in pigment synthesis is catalyzed by other proteins [71]. NRPSs are key players in the synthesis of natural products and provide a modular assembly line by maintaining reaction intermediates covalently bound on the same phosphopantetheine prosthetic group [72]. NRPSs are synthesized as apoforms, followed by activation through phophospantetheinlytransfer to generate the holoenzyme. This activation might be mediated by the gene products of *orf19 *and *orf20*, encoding a protein with a phosphoribosyltransferase domain and a protein with homology to phosphoribosylanthranilate isomerase from *Syntrophus aciditrophicus *[73], respectively. Moreover, the NRPS gene region of plasmid pET44827 includes the coding region *orf17 *with deduced amino acid sequence similarity to IndA from *Thermoanaerobacter tengcongensis*, representing an uncharacterized protein involved in the pigment biosynthesis pathway of this species [74]. Upstream of the NRPS gene region, plasmid pET44827 encodes a putative transcriptional regulator of the MarR protein family (*orf21*), showing 53% similarity to the PecS regulatory protein from *E. chrysanthemi *[75]. Interestingly, the PecS protein is a global regulator of various virulence factors and controls the production of the extracellular blue pigment of *E. chrysanthemi *[75,76]. According to this annotation of pET44827 genes, it is most likely that the characteristic black pigment of *C. aurimucosum *ATCC 700975 is synthesized by the concerted action of the NRPS encoded by *orf18 *and an unknown condensase function and that the black pigment molecule is relevant for the proposed pathogenicity of *C. aurimucosum*.

## Discussion

The annotation and bioinformatics analysis of the complete genome sequence of the clinical isolate *C. aurimucosum *ATCC 700975 (*C. nigricans *CN-1) provides detailed insights into the metabolism and lifestyle of this bacterium. The most prominent features of *C. aurimucosum *strains from female urogenital sources are the unusual black pigmentation and their association with complications during pregnancy [12,13]. Due to the black pigmentation and other phenotypic characteristics, these bacteria were initially named *C. nigricans *[14], but were later shown to represent black-pigmented variants of *C. aurimucosum *[12]. Annotation of the gene content of *C. aurimucosum *ATCC 700975 revealed a cluster of coding regions on plasmid pET44827 that is probably involved in the synthesis of the black pigment. The protein encoded by *orf18 *showed similarity to the gene product of *indC *from the phytopathogenic bacterium *E. chrysanthemi *[71]. The *indC *gene of *E. chrysanthemi *codes for a non-ribosomal peptide synthetase that carries out the stepwise synthesis of the blue pigment indigoidine. Reverchon *et al*. demonstrated that the production of indigoidine conferred an increased resistance to oxidative stress and enhanced the tolerance of *E. chrysanthemi *cells to external hydrogen peroxide [71]. The protection of the bacterial cells by indigoidine is probably based on common chemical properties of many pigment molecules that comprise several carbonate double bonds and can therefore act as efficient radical scavengers [71,77]. This protective function of a bacterial pigment might be relevant also for *C. aurimucosum *when colonizing the human vaginal tract.

The healthy human vaginal microflora is mostly dominated by acidophilic lactobacilli that play important roles in preventing disease, such as bacterial vaginosis, by creating a low-pH environment with a product of their metabolism, lactic acid, and by producing hydrogen peroxide [54]. The synthesis of hydrogen peroxide by lactobacilli provides a major protection mechanism against opportunistic pathogens in the female genital tract [78]. Hydrogen peroxide is toxic in two ways, firstly by producing toxic hydroxyl radicals and secondly by combining with the pool of chlorine ions in the vagina to generate chloridium ions [79]. Hence, the colonization of the female genital tract by *C. aurimucosum *may depend on the efficient protection against cellular damage induced by external hydrogen peroxide. Although the genome of *C. aurimucosum *ATCC 700975 contains a standard gene repertoire for defense against oxidative stress (including catalase, superoxide dismutase and alkyl hydroperoxide reductase), efficient protection of *C. aurimucosum *ATCC 700975 cells against reactive oxygen species in the human vagina is additionally achieved by the synthesis of the typical black pigment that might act as radical scavenger. The location of the corresponding genes on plasmid pET44827 explains why black-pigmented and non-pigmented *C. aurimucosum *strains were isolated from clinical specimens. Remarkably, only black-pigmented strains were recovered from female genital sources, pointing towards the pivotal role of pigmentation for the colonization of the vaginal tract. However, when considering complications during pregnancy, such as bleeding, lactobacilli are less efficient in producing hydrogen peroxide, causing increased alkaline conditions in the vagina and thereby permitting the overgrowth of pathogenic microbes [80]. It would be interesting to screen non-pregnant women and sexually active men to see if some of them are colonized with this bacterium.

Although *Corynebacterium *species appeared to be present in the healthy human vaginal microflora [78,81-83] and on the vaginal epithelium [84], *C. aurimucosum *was hitherto not detected in specimens from the genital tract of healthy women. Which pathway of vaginal, cervical and intrauterine infection by *C. aurimucosum *is therefore likely? Information regarding the non-urogenital habitat of *C. aurimucosum *is currently based on only three reports: (i) Three 16S rDNA sequences were detected in specimens from the forearm skin of a healthy male, indicating that *C. aurimucosum *is part of the human skin biota [19]. (ii) A single 16S rDNA sequence was reported in the prostate fluid from a prostatitis patient [85]. (iii) An apparently pigmented *C. aurimucosum *strain, originally named *C. nigricans *AE1-3, was recovered from a fecal sample of a 35-year-old male Japanese volunteer [37]. This isolation of *C. aurimucosum *from a fecal sample suggested that this species (and its pigmented variants) may reside in the human intestine. The specific metabolic features of *C. aurimucosum *ATCC 700975 deduced from the annotation of detected singletons corroborate this microbiological observation. *C. aurimucosum *may therefore gain access to the lower genital tract by contamination, followed by the so-called ascending pathway from the vagina and cervix, which is the most common route of intrauterine infections [80]. However, almost all of the black-pigmented variants of *C. aurimucosum *have been reported from urogenital sources suggesting that it may be the primary niche for this potential pathogen. Indeed, its capability to (i) attach to mucosal surfaces through pili, (ii) make biofilms, and (iii) utilize aromatic compounds and L-histidine suggests that it is fully equipped to invade vaginal mucosa. When viewed in the context of its consistent isolation from pregnant women during complications of pregnancy, it is reasonable to assume that it plays an important role there.

In the gut ecosystem, aromatic amines and aromatic acids can be a frequent carbon and energy source for microbial growth when oxygen is available for the enzymatic ring cleavage reaction [39]. As oxygen molecules are present in close proximity to epithelial cells, where they pass from the blood through the epithelium, bacterial cells attached to the epithelium can assimilate such molecules and can thus benefit from the presence of oxygen [40]. The adherence of *C. aurimucosum *to epithelial cells is apparently mediated by a pilus structure of the SpaH type, consisting of the major pilin SpaH and the minor pilins SpaI and SpaG. Adherence of *C. diphtheriae *to pharyngeal epithelial cells can be mediated also by the minor pilin SpaB of the structurally related SpaABC pilus [57]. The SpaB protein is covalently anchored to the cell wall to provide tight contact between the bacterial cell and the host tissue in the absence of a pilus shaft. Taking into consideration a similar functioning of the SpaI subunit of the SpaH pilus, adherence of *C. aurimucosum *cells to host tissue may also occur in the absence of a pilus shaft. Adhesive pili can mediate the initial contact to host cell receptors, with cell-wall-linked pilins supporting the formation of an intimate zone of adhesion. This close contact between the bacterium and host cells enables additional ligand-receptor interactions and, in the case of pathogenic interactions, the efficient delivery of virulence factors [58]. Furthermore, the adherence of *C. aurimucosum *to epithelial cells may result in the formation of biofilms. The cross-linking of bacterial cells can be mediated by the G5 domains of the accumulation-associated protein Aap that is encoded on the *C. aurimucosum *ATCC 700975 chromosome and can probably interact with *N*-acetylglycosamine residues present in the peptidoglycan of the bacterial cell wall [59].

## Conclusions

The annotation of the gene repertoire provides the attractive hypothesis to consider *C. aurimucosum *as resident of the human gut system that manages to reach the lower female genital tract and to establish in the vaginal environment as a contaminant. The close proximity of the vagina to the rectum raises the possibility of microbial colonization of the female genital tract in a continuous process [86]. Another possibility is that black-pigmented strains are sexually transmitted from infected men to some women who engage in sex during pregnancy. Indeed, a *C. aurimucosum*-like 16S rDNA sequence has been reported from a prostatitis case [85]. Further studies are necessary to confirm if *C. aurimucosum *is frequently found in sexually active men. When *C. aurimucosum *is equipped with appropriate protection mechanisms against the vaginal hydrogen peroxide produced by acidophilic lactobacilli, it can apparently establish in the vagina and cervix [11-13] and probably ascend to the amniotic cavity and the fetus. Bacterial infection by contamination is thereby followed by colonization that might cause distinct maternal or fetal responses [79,87]. The results of the *C. aurimucosum *genome project may help to clarify the proposed role of this bacterium as urogenital pathogen in pregnant women and to prevent preterm delivery that is a leading cause of infant morbidity and mortality in the U.S.A. and Europe [79]. If the clinical diagnosis of *C. aurimucosum *infection and medical intervention is timely, the prognosis for pregnant women with urogenital infections caused by *C. aurimucosum *appears to be reasonably good, since several antibiotics turned out recently to be useful for the treatment of infections caused by this organism [88].

## Methods

### Bacterial strain and growth conditions

*C. aurimucosum *ATCC 700975 (DSM 44827, CCUG 48176, CIP 107436), formerly designated *C. nigricans *CN-1 [10], was obtained as a lyophilized culture from DSMZ (Braunschweig, Germany). The strain was originally isolated from the urogenital tract of a 34-year-old woman who experienced a spontaneous abortion during month six of pregnancy [10]. *C. aurimucosum *was routinely cultured on solid BYT complex medium [89] at 37°C. For the preparation of genomic DNA samples, *C. aurimucosum *was grown for 24 h in liquid BYT medium supplemented with 1.25% (w/v) glycine.

### Sequencing of the *C. aurimucosum *ATCC 700975 genome

Genomic DNA of *C. aurimucosum *ATCC 700975 was purified from 20-ml aliquots of an overnight culture by an alkaline lysis procedure [90]. The published protocol was sligthly modified as follows: (i) 20-ml aliquots of *C. aurimucosum *cells were incubated in a 30 mg ml^-1 ^lysozyme solution at 37°C for 1 h. (ii) The harvested cells were lysed in 0.7 ml 10% (w/v) SDS solution at 37°C for 15 min. A total of 5 μg of purified genomic DNA was used for constructing a single-stranded template DNA (sstDNA) library. The concentration of the sstDNA library was quantified by using the Agilent RNA 6000 Nano Kit. Preparation of the sstDNA library and subsequent DNA sequencing were performed according to manufacturer's protocols (Roche Applied Science). Pyrosequencing of *C. aurimucosum *DNA was carried out with the Genome Sequencer FLX System (Roche Applied Science). The sequence reads were assembled with the GS Assembler Software (Version 1.1.02). Using the default cutoff of 500 bases for the classification of contigs, 73 large contigs (≥ 500 bases) and 40 small contigs were assembled to give a total size of 2,744,972 bases.

A fosmid library with a 7-fold coverage of the *C. aurimucosum *ATCC 700975 genome was constructed in *E. coli *EPI300 cells with the Copy Control Fosmid Library Production Kit and the pCC1FOS fosmid vector (Biozym Scientific). Terminal insert sequences from fosmid library clones were used to build a scaffold for the genome sequence assembly. The terminal insert sequences and the assembled genomic contigs obtained by pyrosequencing were combined by means of the Consed program [15]. To close the remaining gaps in the *C. aurimucosum *chromosome, additional Sanger reads were generated on purified DNA of selected "linking fosmids". The assembled contigs of plasmid pET44827 were bridged by PCR products obtained with Phusion DNA polymerase (Finnzymes) on genomic template DNA. All primers used in this study were synthesized by Eurofins MWG Operon.

### Bioinformatics analysis and annotation of the genome sequence

To verify the assembly of the *C. aurimucosum *ATCC 700975 genome sequence, the paired-end sequence information obtained from fosmid clones was used to generate a genomic map with the BACCardI program [91]. Automated annotation of the *C. aurimucosum *genome sequence was carried out with the bacterial genome annotation platform GenDB [16] as described earlier [92], followed by manual curation. Proteins that are orthologous in completely sequenced pathogenic corynebacteria, *C. diphtheriae*, *C. jeikeium*, *C. urealyticum*, and *C. kroppenstedtii *[30,31,38,92], were deduced by bidirectional best BLASTP hits [22]. Two proteins were considered as orthologs if BLASTP matches with at least 30% sequence identity and a minimum coverage of 50% of the query sequence length were detected in both directions. The orthologous pair of proteins have to represent additionally the best BLASTP hits for the respective query sequence in the target genome. Genes were added to the core genome of completely sequenced pathogenic corynebacteria if orthologs of the encoded proteins were detected in the complete set of selected genomes. If none of the BLAST hits met the criteria defined above in any other genome, the genes were classified as singletons (specific) for *C. aurimucosum *ATCC 700975. Gene content and comparative metabolic analyses were accomplished by the computer programs EDGAR and CARMEN [35,36]. The annotated genome sequence of *C. aurimucosum *ATCC 700975 was submitted to the GenBank/EMBL database with accession numbers CP001601 (chromosome) and FM164414 (plasmid pET44827).

## Authors' contributions

ET coordinated the project, sequenced the *C. aurimucosum *genome, carried out the manual annotation and prepared the manuscript. KHG purified the genomic DNA. RS, AT, PV, and WA participated in genome sequencing. SG and SSB supported the gap closure process. JS, TB, JB, BL, and AG provided bioinformatics support for the genome annotation. AP, SKS, and AT supervised the work and participated in data evaluation. All authors read and approved the final version of the manuscript.
